# Prostaglandins in human mammary cancer.

**DOI:** 10.1038/bjc.1984.73

**Published:** 1984-04

**Authors:** D. M. Watson, R. W. Kelly, R. A. Hawkins, W. R. Miller

## Abstract

Prostaglandins E2 and F2 alpha (PGE2 and PGF2 alpha) were measured by Gas Liquid Chromatography-Mass Spectrometry (GLC-MS) in extracts of 100 human mammary carcinomas. All tumours contained measurable amounts of both prostaglandins but wide variations between individual tumours were observed. Values for PGE2 ranged from 7 to 762 ng g-1 tissue with a median of 100 ng g-1 tissue. Values for PGF2 alpha ranged from 3 to 475 ng g-1 tissue (median 60 ng g-1 tissue). There was a highly significant positive correlation between amounts of the 2 prostaglandins in individual tumours. Amounts of both PGE2 and PGF2 alpha were not significantly related to the menopausal status of the patients or the presence of oestrogen and progesterone receptors.


*Br. J. Cancer* (1984), **49**, 459–464

### Prostaglandins in human mammary cancer

D.M.A. Watson[1], R.W. Kelly[2], R.A. Hawkins[1] & W.R. Miller[1]

[1]*University Department of Clinical Surgery, Royal Infirmary;* [2]*Centre for Reproductive Biology, Chalmers Street, Edinburgh, UK*

**Summary** Prostaglandins $E_2$ and $F_{2\alpha}$ ($PGE_2$ and $PGF_{2\alpha}$) were measured by Gas Liquid Chromatography – Mass Spectrometry (GLC-MS) in extracts of 100 human mammary carcinomas. All tumours contained measurable amounts of both prostaglandins but wide variations between individual tumours were observed. Values for $PGE_2$ ranged from 7 to 762 ng g$^{-1}$ tissue with a median of 100 ng g$^{-1}$ tissue. Values for $PGF_{2\alpha}$ ranged from 3 to 475 ng g$^{-1}$ tissue (median 60 ng g$^{-1}$ tissue). There was a highly significant positive correlation between amounts of the 2 prostaglandins in individual tumours. Amounts of both $PGE_2$ and $PGF_{2\alpha}$ were not significantly related to the menopausal status of the patients or the presence of oestrogen and progesterone receptors.

Human mammary cancers appear to produce prostaglandin-like material, and this may be involved in tumour growth and metastases (Bennet *et al.*, 1977; Rolland *et al.*, 1980). Most investigations have, however, employed bioassay or radioimmunoassay techniques which do not definitively identify prostaglandins. A single study has reported on determinations performed by GLC-mass spectrometry but the number of tumours examined was relatively small (Stamford, 1983). The present investigation represents a study of 100 human mammary cancers in which $PGE_2$ and $PGF_{2\alpha}$ have been identified and quantitated by GLC-mass spectrometry. Values of prostaglandins have then been related to tumour steroid receptor status.

### Materials and methods

### *Tumours*

Tumour was obtained from 100 women with carcinoma of the breast. These patients comprised 15 premenopausal, 10 perimenopausal (within 5 years of the last menstrual period) and 75 post menopausal women. Tumour was removed at mastectomy or biopsy from the primary cancer (in 88 women), by biopsy of invaded lymph node (in 9 cases) or secondary recurrences (in 3 cases). For comparative purposes, material was also obtained from benign fibroadenoma of the breast in 5 women and from histologically normal breast tissue in 3 women. This material was placed on ice and immediately transferred to the laboratory. Following removal of tissue for histopathological diagnosis, the remaining material was dissected free of extraneous fat and divided for prostaglandin and steroid receptor assays.

Correspondence: D.M.A. Watson.

Received 18 December 1983; accepted 19 January 1984.

### *Measurement of prostaglandins*

*Formation of derivatives* Tumour samples were weighed and homogenized in ethanol (2.5 ml). To 1 ml duplicates of each sample were added 20 ng of the internal standards, (20-ethyl $PGF_{2\alpha}$ and 20-methyl $PGE_2$.). Oximes were formed by adding hydroxylaminehydrochloride solution (50 mg ml$^{-1}$) in sodium acetate buffer (3 mol l$^{-1}$, pH 5.2) and heating for 30 min at 60°C. The samples were extracted and purified using a C18 Seppak column, washed with 10 ml iso-octane and then 10 ml 50% methanol to remove neutral lipids. The prostaglandins were then eluted with 90% methanol (10 ml). This fraction was evaporated to dryness and the residue dissolved in ethyl acetate:ethanol (1:1 v/v), transferred to a small flat-bottomed tube and methylated with 100 $\mu$l diazomethane solution. Excess diazomethane and solvent were then evaporated. The residue was further derivatized to the t-butyldimethylsilyl ether by adding 65 $\mu$l 2M-butyldimethylchlorosilane and 65 $\mu$l 4M-imidazole (both in dimethylformamide). After mixing, this solution was transferred to a narrow glass tube which was then sealed and heated for 30 min at 130°C. The excess reagents were removed from this mixture by eluting the derivative with 3 ml hexane:ethylacetate (3:1 v/v) from a Sephadex LH-20 column. The solvent was evaporated and 20 $\mu$l toluene added to the derivative. About one twentieth of this mixture was injected into the gas chromatograph.

© The Macmillan Press Ltd., 1984

*Gas chromatography – mass spectrometry* Samples were analysed with a Erba Science gas chromatograph coupled through an all-glass jet separator to a V.G.305 mass spectrometer. An open tubular column coated with $SE_{30}$ and 12 m long (SGE Ltd. London) was used. The flow rate of helium was $5\,ml\,min^{-1}$. The temperatures were maintained as follows: gas-chromatography column 280°C; separator and lines 260°C; source block 270°C. The signal was processed by a 2150 data system to allow separate GC traces for each.

For analysis of $PGF_{2\alpha}$ the mass used was m/z 653 and 681 and for $PGE_2$, m/z 666 and 680. The ions measured were the M-57 ions resulting from the loss of a t-butyl radical from the molecular ion. Quantitation was achieved by comparing the areas of the sample peak with those of the corresponding standards. Procedural losses were corrected by monitoring the recovery of the internal standards, 20 methyl PGE2 (m/z 680) and 20 ethyl $PGF_{2\alpha}$ (m/z 681). The intra-assay precision was 13%; values for interassay precision were 18 and 21% for $PGF_{2\alpha}$ and $PGE_2$ respectively.

*Oestrogen receptors*

Concentration of oestrogen receptors was determined by saturation analysis (Hawkins *et al.*, 1975). Tumour cytosol was incubated overnight at 4°C with [$^3$H] 17 $\beta$-oestradiol. Separation of free and bound steroid was by addition of dextran-coated charcoal; the bound fraction was measured by liquid scintillation counting. Concentration of receptors was determined by Scatchard analysis (Scatchard, 1949). Activities in excess of $5\,fmol\,mg^{-1}$ cytosol protein were designated receptor positive.

*Progestogen receptors*

Cytosol was incubated with a fixed concentration of [$^3$H] Organon-2058 (0.22 nM) and varying concentrations of non-radioactive Organon-2058 (0.22–11.1 nM) with overnight binding at 0°C and separation of free and bound hormone by charcoal adsorption (Hawkins *et al.*, 1981). Activities in excess of $10\,fmol\,mg^{-1}$ cytosol protein were designated receptor positive.

*Protein concentration* in the cytosol was determined by the method of Bradford (1976).

*Statistical analysis*

Non-parametric tests (i.e. Wilcoxon's Rank Test and Spearman's Rank correlation) were used throughout these studies.

### Results

Measurable amounts of $PGE_2$ and $PGF_{2\alpha}$ were

**Figure 1** Levels of $PGE_2$ and $PGF_{2\alpha}$ in 100 human breast cancers. Lines represent the median values.

detected in all carcinomas. Levels in individual tumours are shown in Figure 1. Range of values for $PGE_2$ was from 7 to $762\,ng\,g^{-1}$ tissue with a median value of 100. The corresponding range for $PGF_{2\alpha}$ in the same samples was from 3 to $475\,ng\,g^{-1}$ tissue (median 60). Values in a group of 5 fibroadenomas ranged from $2–19\,ng\,g^{-1}$ tissue (median 9) for $PGE_2$ and $6–14\,ng\,g^{-1}$ tissue (median 12) for $PGF_{2\alpha}$ and those for 3 specimens of histologically normal breast were $7–14\,ng\,g^{-1}$ tissue for $PGE_2$ and $5–18\,ng\,g^{-1}$ for $PGF_{2\alpha}$.

Within the group of breast cancers, there was a highly significant correlation between amounts of $PGE_2$ and $PGF_{2\alpha}$ (Figure 2). (Spearman's Rank correlation coefficient = 0.543 $P<0.001$).

There was no significant difference between prostaglandin levels in primary tumours, lymph nodes and secondary recurrences (Data not shown). Nor were there significant differences in amounts of either $PGE_2$ or $PGF_{2\alpha}$ in pre, peri and postmenopausal patients (Figure 3).

Oestrogen receptor activity was detected in 68 tumours (68%) and the relationship between the presence of receptors and prostaglandin content is

**Figure 2** The relationship between levels of $PGE_2$ and $PGF_{2\alpha}$. Line is that of linear correlation. Correlation coefficient by Spearman Rank test 0.543 $P<0.001$.

**Figure 3** Levels of prostaglandins in tumours from pre, peri and postmenopausal patients. Lines represent median values. No significant differences between the groups by Wilcoxon Rank Test.

shown in Figure 4. The median value for both $PGE_2$ and $PGF_{2\alpha}$ was higher in tumours with oestrogen receptors as compared to those without receptors, but the difference between the two groups failed to reach statistical significance. ($P<0.1$ and $P<0.2$, respectively by Wilcoxon Rank Sum Test).

**Figure 4** Levels of prostaglandins in oestrogen receptor positive (+) and negative (−) tumours. Lines represent median values. No significant difference between the groups by Wilcoxon Rank Test.

Progesterone receptors were detected in 34 of the 93 tumours investigated (37%). There was no significant difference between either $PGE_2$ or $PGF_{2\alpha}$ levels in progesterone receptor positive and negative tumours (Figure 5). ($P<0.8$ and $P<0.9$ respectively by Wilcoxon Rank Sum Test).

### Discussion

To date, the evidence that human breast cancers contain significant amounts of prostaglandins has been based on data from either bioassay or radioimmunoassay. These techniques, although of value, do not accurately identify different prostaglandins. In the present study, therefore, we have used the more definitive technique of GLC-mass spectrometry which has previously only been employed for prostaglandin measurements in a small group of breast cancers (Stamford, 1983).

**Figure 5** Levels of prostaglandins in progesterone receptor positive (+) and negative (−) tumours. Lines represent median values. No significant difference between the groups by Wilcoxon Rank Test.

It is almost impossible to measure "in situ" levels of prostaglandins within tissue preparations because prostaglandins are not stored in cells but are synthesised rapidly in response to stimuli. Biopsy, processing and homogenization of tumour specimens represent stimuli which would result in production of large amounts of prostaglandins. Determination of prostaglandins in tumours can, therefore, only be directed towards assessing the potential for prostaglandin production rather than measurement of endogenous levels (Green, 1979). Two types of technique have been adopted in this respect. Tumour may be homogenized directly in ethanol to obtain "basal" levels or incubated with or without added precursor to determine "synthesised" levels. It is not clear which technique more accurately reflects tumour potential for producing a local environment of prostaglandins or indeed if either reflects "in situ" activity. "Basal" levels will include both the normal content of tumour cells and material synthesized between biopsy and inactivation of synthetic ensymes during homogenisation (Bennet, 1982). The level of prostaglandin 'synthesized" will depend critically on precautions taken to protect the highly labile prostaglandin synthetase system and addition of arachidonate substrate may not mimic tissue levels of precursor. We have, therefore, chosen to measure basal levels of prostaglandins in the present study because this represents the least complicated and most practical method of studying prostaglandins in a large number of cancers.

Using these techniques, measurable amounts of $PGE_2$ and $PGF_{2\alpha}$ were detected in all tumours. Prostaglandins were identified on the basis of their molecular ions (at m/z 666 $PGE_2$, m/z 653 for $PGF_{2\alpha}$), retention times and characteristic peaks. Amounts of $PGE_2$ varied from 7 to 762 ng g$^{-1}$ tissue and those of $PGF_{2\alpha}$ from 3 to 475 ng g$^{-1}$ tissue. These amounts are comparable to concentrations demonstrated by other methods (Bennet, 1982) and were higher than those found in benign and normal breast. A strong positive correlation was detected between levels of the two prostaglandins in individual breast cancers as has been previously observed (Fulton *et al.*, 1982). Although the variation in the amounts of both prostaglandins was large, we have been unable to identify factors accounting for this variation. Levels of tumour prostaglandins appeared to be uninfluenced by menopausal status of the patient, site of tumour biopsy or whether cytosolic steroid receptors were present.

The finding of no significant difference in tumour prostaglandin levels between pre, peri and post-menopausal patients agrees with the results of Rolland *et al.* (1980) who measured "synthesized" levels of prostaglandins in microsomal preparations. Fulton *et al.* (1982) failed to show an association with $PGF_{2\alpha}$ but reported significantly raised $PGE_2$ levels in postmenopausal women. It has been suggested that oestrogen receptor positive tumours synthesize greater amounts of prostaglandins (Campbell *et al.*, 1982). Others have been unable to show such a correlation (Rolland *et al.*, 1980; Bennet, 1982). However, in the study which reported a significant correlation, it was necessary to make a correction for tumour cellularity before the association became apparent. In the present study, a significant correlation was apparent between tumour cellularity and $PGE_2$ but not $PGF_{2\alpha}$ (Figure 6). However, multiple regression analysis of $PGE_2$ and $PGF_{2\alpha}$ on both oestrogen receptor and cellularity showed that oestrogen receptor had no significant effect on prostaglandins for given levels of cellularity (for $PGE_2$ $t=0.36$, for $PGF_{2\alpha}$ $t=1.12$). Indeed, correcting the results for tumour cellularity may be misleading because this would be based on the assumption that tumour cells within the biopsy were solely responsible

**Figure 6** Levels of prostaglandins in tumours with different grades of cellularity. Cellularity was assessed as described by Hawkins *et al.* (1981) using a 4 point scale for both the proportion of tumour in the tissue specimen and for the proportion of malignant cells within the tumour itself. These are expressed as a product of the two scores to give a range of 1–16. (Score 1–4 therefore represents tumours with a low cellularity index, those with 13–16 the highest) $PGE_2$ correlates significantly with cellularity ($t=0.14$, $P<0.05$, by Kendal Rank Test). $PGF_{2\alpha}$ did not correlate significantly with cellularity ($t=0.08$).

whereas other cell types such as macrophages, lymphocyctes, plasma cells and fibroblasts may also be producing prostaglandins. We have, therefore, preferred to express our results in terms of prostaglandins extracted per weight of tumour tissue.

Variation in prostaglandins extractable from breast cancers may be due to non-specific, non-tumour factors such as the time interval between the biopsy and extraction, and the degree of trauma produced in obtaining the tumour samples. Enzymes associated with prostaglandin synthesis are particularly labile (Egan *et al.*, 1978) and it is essential to minimise any delay in tumour processing. Substantial amounts of prostaglandins may be generated in response to trauma (Green, 1979) and variation in degree of tissue trauma might be expected to be associated with differences in prostaglandins levels. In practice, it is difficult to eliminate tissue trauma as even gentle handling may stimulate biosynthesis of prostaglandins. In this series of tumours, clinical and pathological considerations determined the degree of mechanical trauma to which the specimens were subjected. However, no significant differences were detected between prostaglandins extracted from samples obtained at biopsy and those from mastectomy specimens.

It has been suggested that tumour prostaglandins are associated with prognosis and pattern of metastatic spread of breast cancer. At the present time, data from this study cannot be assessed for these parameters. Most tumours were obtained from patients with early breast cancer who have, as yet, only short follow-up. The absence of positive correlations with other factors of known prognostic value such as steroid receptors and lymph node status could mean that prostaglandins are either of independent significance or unrelated to prognosis. Valid assessment of the data will only be possible when further follow-up of the patients is available.

We would like to thank Professor A.P.M. Forrest for allowing us to study material from patients under his care, Dr J.M.J. Dixon for assessing the cellularity of each tumour and Dr R.A. Elton, Department of Medical Statistics and Computing for statistical analysis of the results.

### References

BENNET, A. (1982). Prostaglandins: relationship to breast cancer and its spread. In: *Endocrine Relationships in Breast Cancer*, (Ed. Stoll), London, W. Heinemann, p. 156.

BENNAT, A., CHARLIER, E.M., McDONALD, A.M., SIMPSON, J.S., STAMFORD, I.F. & ZEBRO, T. (1977). Prostaglandins and breast cancer. *Lancet*, **ii**, 124.

BRADFORD, M.M. (1976). A rapid and sensitive method for the quantitation of microgram quantities of protein utilizing the principle of protein-dye binding. *Analyt. Biochem.*, **72**, 248.

CAMPBELL, F.C., HAYNES, J., EVANS, D.F., MUMFORD, R.W., BLAMEY, R.W., ELSTON, C.W. & NICHOLSON, R.I. (1982). Prostaglandin E2 synthesis by tumour epithelial cells and oestrogen receptor status of primary breast cancer. *Langenbecks Arch. Chir.*, **357**, 209.

EGAN, R.W., PAXTON, J. & KUEHL, F.A. (1978). Mechanism for irreversible self-deactivation of prostaglandin synthetase. *J. Biol. Chem.*, **251**, 7329.

FULTON, A., ROI, L., HOWARD, L., RUSSO, J., BROOKS, S. & BRENNAN, M.J. (1982). Tumour-associated prostaglandins in patients with primary breast cancer; relationship to clinical parameters. *Breast Cancer Res. Treat.*, **2**, 331.

GREEN, K. (1979). Determination of prostaglandins in body fluids and tissues. *Acta. Obstet. Gynecol. Scand.* (*Suppl.*) **87**, 15.

HAWKINS, R.A., HILL, A. & FREEDMAN, B. (1975). A simple method for the determination of oestrogen receptor concentrations in breast tumours and other tissues. *Clin. Chem. Acta*, **64**, 203.

HAWKINS, R.A., BLACK, R., STEELE, R.J.C., DIXON, J.M.J. & FORREST, A.P.M. (1981). Oestrogen receptor concentration in primary breast cancer and axillary node metastases. *Breast Cancer Res. Treat.*, **1**, 245.

ROLLAND, P.H., MARTIN, P.M., JAQUEMIER, J., ROLLAND, A.M. & TOGA, M. (1980). Prostaglandin in human breast cancer: Evidence suggesting that an elevated prostaglandin production is a marker of high metastatic potential for neoplastic cells. *J. Natl Cancer Inst.*, **64**, 1061.

SCATCHARD, G. (1949). The attraction of protein for small molecules and ions. *Ann. N.U. Acad. Sci.*, **57**, 660.

STAMFORD, I.F. (1983). Identification of arachidonate metabolites in normal, benign and malignant human mammary tissues. *J. Pharm. Pharmacol.*, **35**, 48.


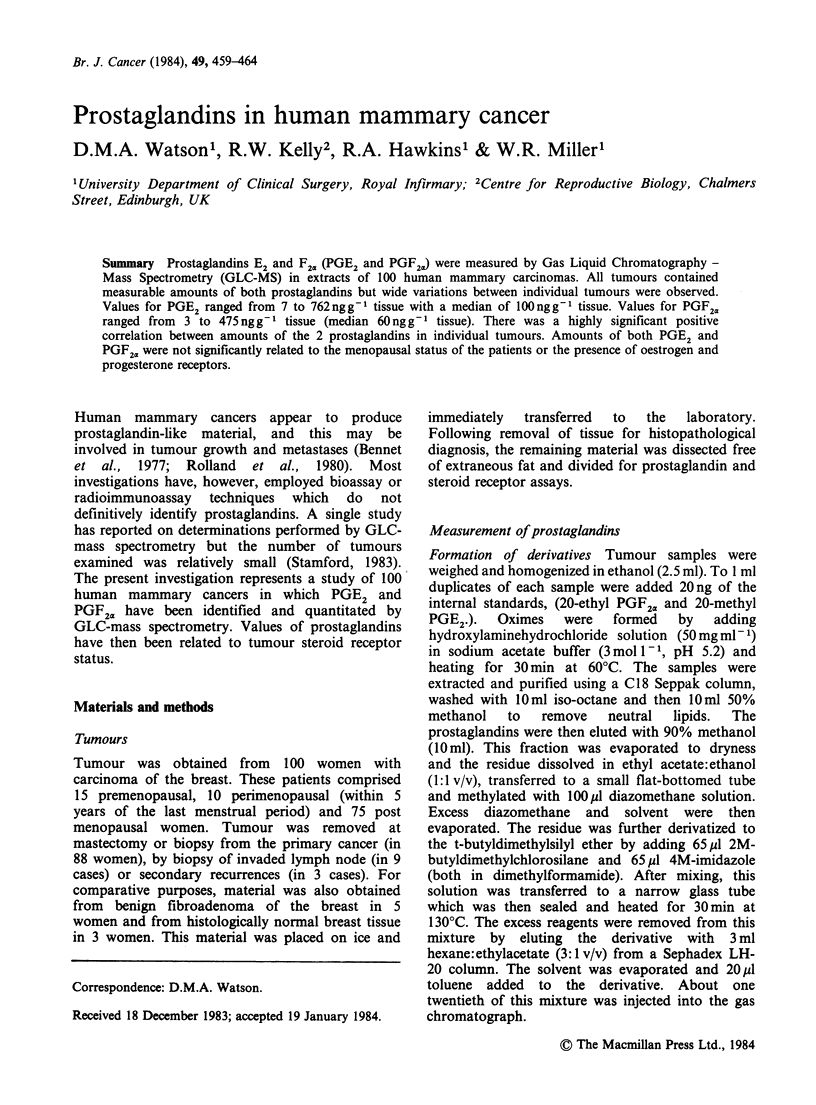

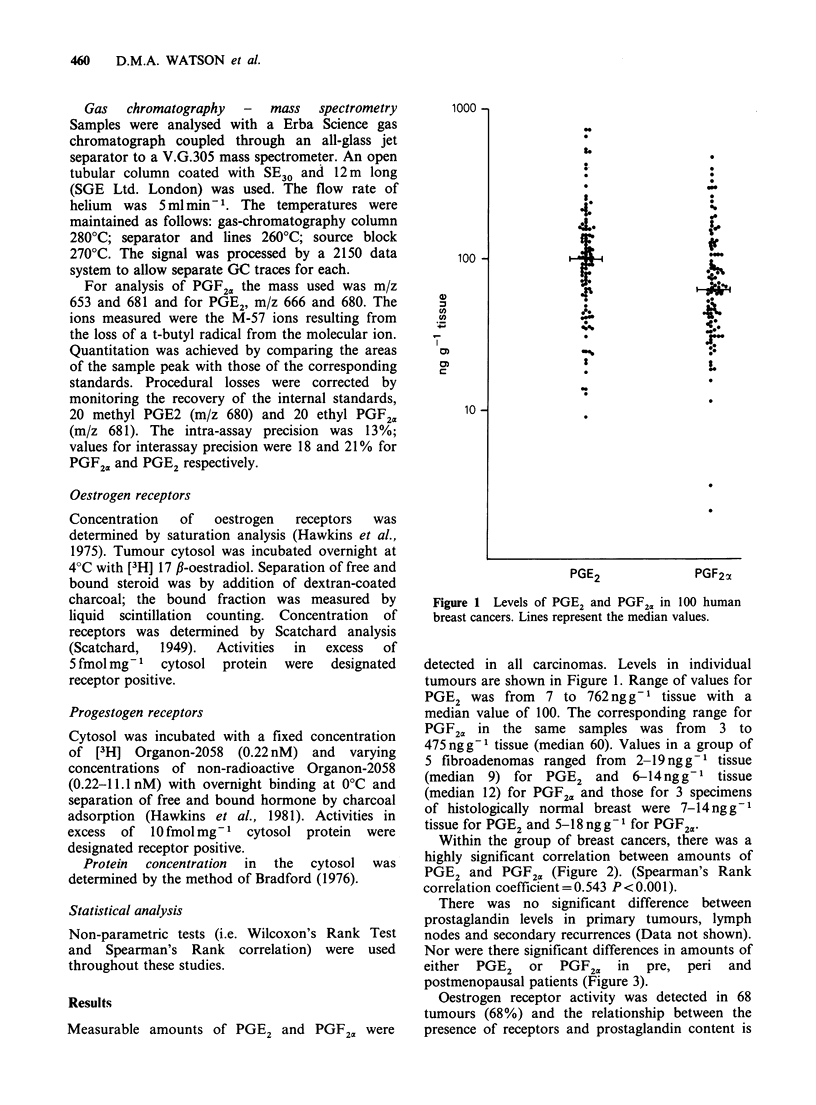

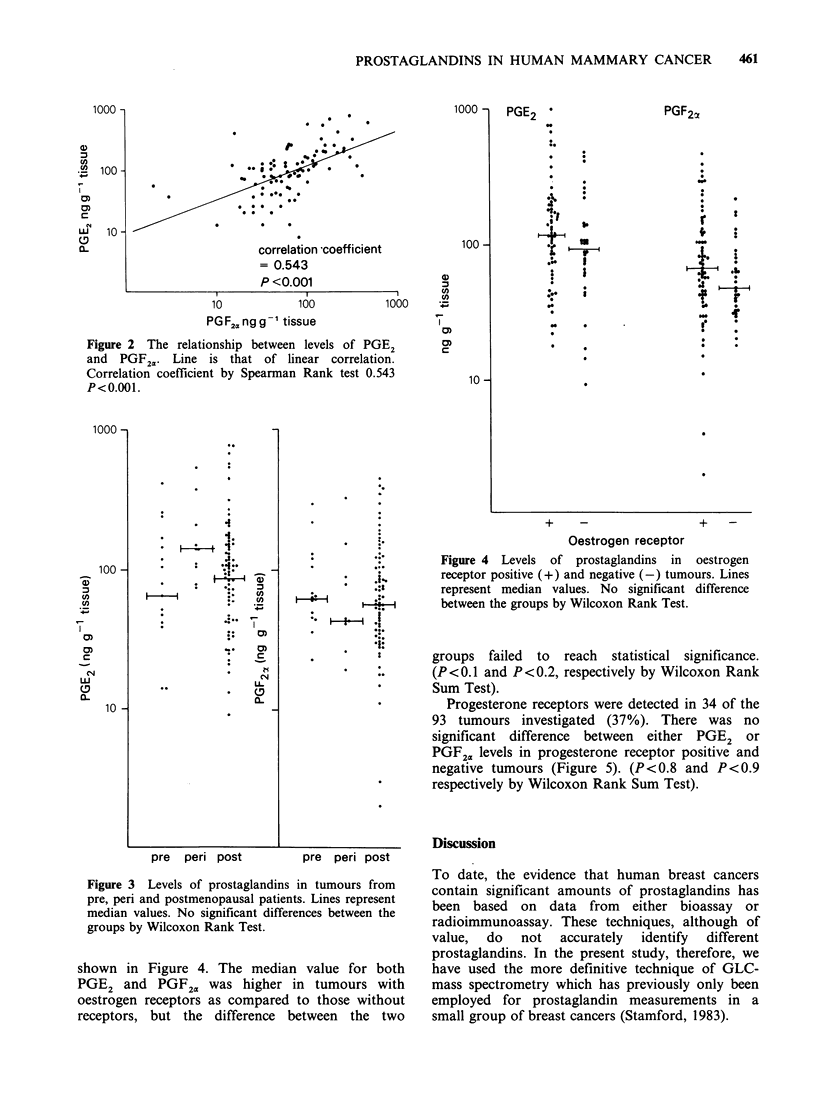

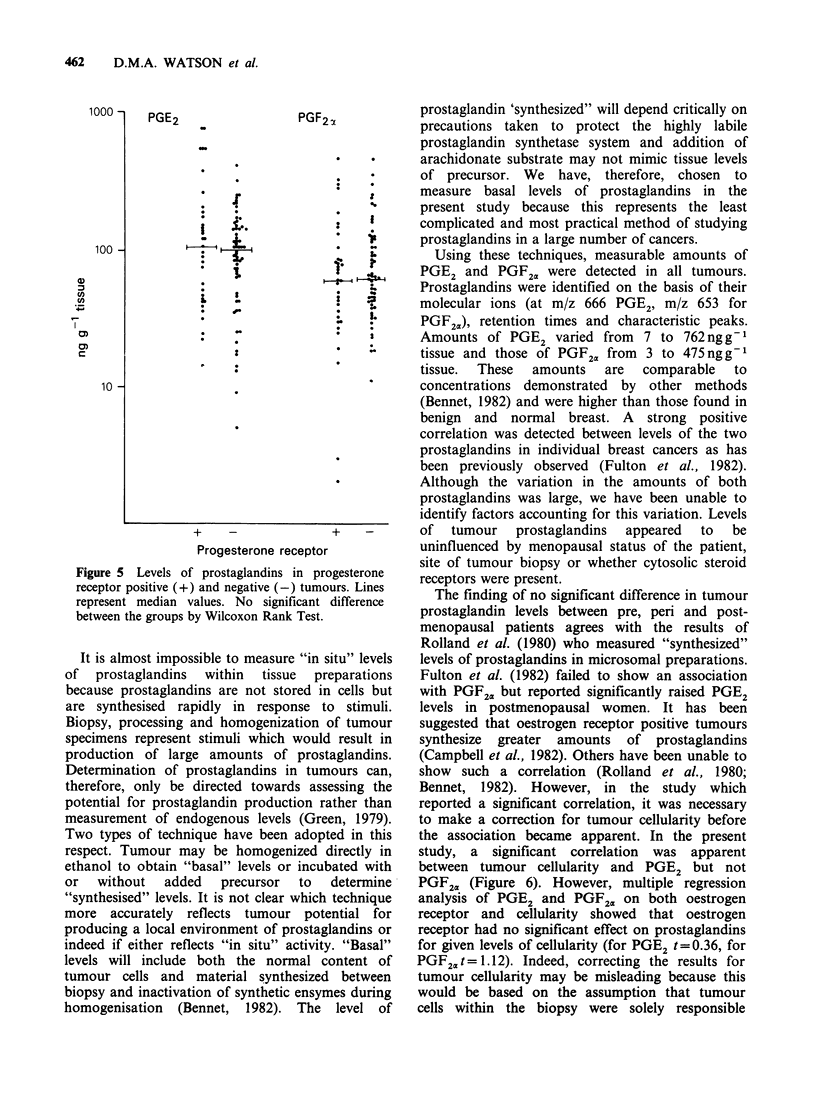

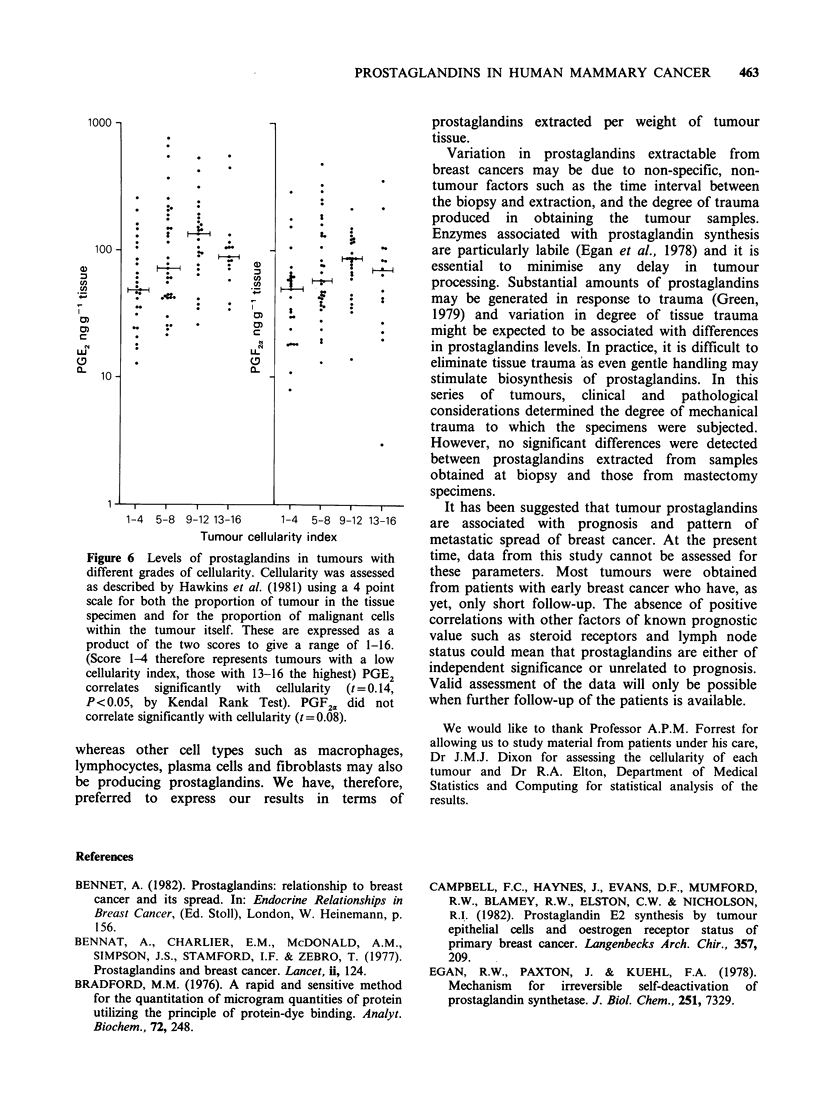

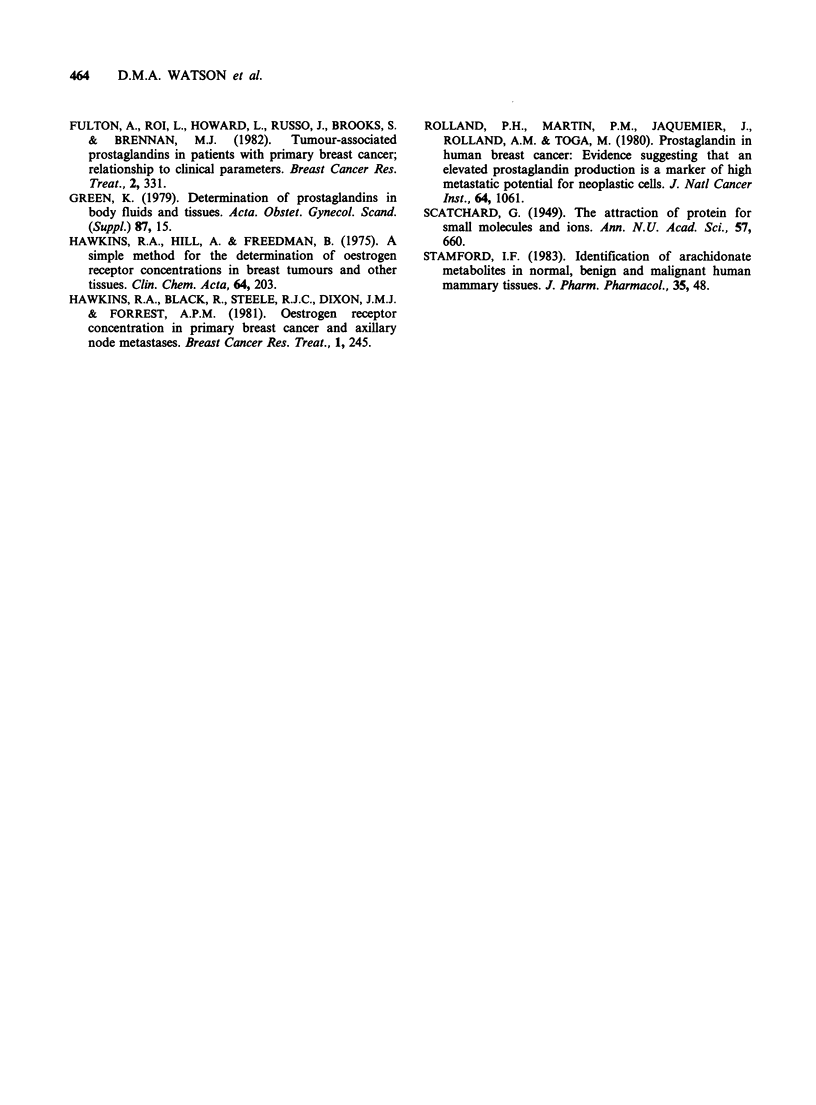


## References

[OCR_00660] Bradford M. M. (1976). A rapid and sensitive method for the quantitation of microgram quantities of protein utilizing the principle of protein-dye binding.. Anal Biochem.

[OCR_00674] Egan R. W., Paxton J., Kuehl F. A. (1976). Mechanism for irreversible self-deactivation of prostaglandin synthetase.. J Biol Chem.

[OCR_00688] Gréen K. (1979). Determination of prostaglandins in body fluids and tissues.. Acta Obstet Gynecol Scand Suppl.

[OCR_00699] Hawkins R. A., Black R., Steele R. J., Dixon J. M., Forrest A. P. (1981). Oestrogen receptor concentration in primary breast cancer and axillary node metastases.. Breast Cancer Res Treat.

[OCR_00693] Hawkins R. A., Hill A., Freedman B. (1975). A simple method for the determination of oestrogen receptor concentrations in breast tumours and other tissues.. Clin Chim Acta.

[OCR_00655] Ing R., Petrakis N. L., Ho J. H. (1977). Unilateral breast-feeding and breast cancer.. Lancet.

[OCR_00705] Rolland P. H., Martin P. M., Jacquemier J., Rolland A. M., Toga M. (1980). Prostaglandin in human breast cancer: Evidence suggesting that an elevated prostaglandin production is a marker of high metastatic potential for neoplastic cells.. J Natl Cancer Inst.

[OCR_00718] Stamford I. F., Carroll M. A., Civier A., Hensby C. N., Bennett A. (1983). Identification of arachidonate metabolites in normal, benign and malignant human mammary tissues.. J Pharm Pharmacol.

